# Weighty Matters: The Obesity–Thyroid Nodule Connection Unveiling the Impact of Obesity on Thyroid Cancer Risk

**DOI:** 10.3390/medicina59091658

**Published:** 2023-09-14

**Authors:** Saad M. Alqahtani, Bassam A. Altalhi, Yousef S. Alalawi, Areej A. AlFattani, Saif S. Al-Sobhi

**Affiliations:** 1 Department of Surgery, College of Medicine, Majmaah University, Al-Majmaah 11952, Saudi Arabia; 2 Department of Surgery, King Fahad Armed Forces Hospital, Jeddah 21159, Saudi Arabia; dr.bassam.ayesh@gmail.com; 3 Department of Surgery, King Salman Armed Forces Hospital Northwestern Region, Tabuk 71411, Saudi Arabia; yalalawi@hotmail.com; 4 Department of Biostatistics, Epidemiology, and Scientific Computing, King Faisal Specialist Hospital and Research Centre, Riyadh 11211, Saudi Arabia; a.alfattani1430@gmail.com; 5 Department of Surgery, King Faisal Specialist Hospital and Research Center, Riyadh 11211, Saudi Arabia; saifalsobhi@hotmail.com

**Keywords:** thyroid nodule, atypia of undetermined significance/follicular lesion of undetermined significance, indeterminate nodule, obesity, thyroid neoplasms

## Abstract

*Background and Objectives:* The effect of obesity on the development/progression of thyroid nodules with uncertain cytology is unknown. Therefore, our objective was to assess the role of body mass index (BMI) in predicting malignancy in patients with atypia of undetermined significance/follicular lesion of undetermined significance (AUS/FLUS) nodules. *Materials and Methods:* We retrospectively analyzed 113 patients with available BMI data and final histopathology of benign or differentiated thyroid cancer. Patients were classified into four groups based on BMI: <18.5 (underweight), 18.5–24.9 (normal weight), 25–29.9 (overweight), and ≥30 (obesity) kg/m^2^. The association between risk of malignancy and BMI was examined for all data and subgroups based on nodule size, sex, and age. *Results:* Overall, 44.2% were obese, 36.3% were ≥45 years, and 75.4% were women. Final pathological results showed malignant nodules in 52 patients (46%) and benign nodules in 61 patients (54%) (mean age: 41 ± 11.6 vs. 39.9 ± 11.7 years; *p* = 0.62). Men had more malignant nodules than benign nodules (32.7% vs. 16.4%, *p* < 0.05). Overall, no significant correlation was identified between the risk of thyroid cancer and BMI, and the risk of malignancy was not significantly different between obese men and women (*p* = 0.4). However, in individuals with BMI < 30 kg/m^2^ (non-obese group), malignant nodules were more frequent in men than in women (71% vs. 41%, *p* = 0.04). No significant difference was observed in mean nodule size between the benign and malignant groups. Furthermore, BMI was not related to increased risk of malignancy in multiple logistic regression models using all data, even after controlling for confounding variables (odds ratio, 0.99, 95% confidence interval: 0.93–1.06, *p* = 0.87) or when stratifying by sex. *Conclusions:* Our study showed no correlation between obesity and thyroid cancer in patients with AUS/FLUS. Moreover, men had more malignant nodules than benign nodules. Further well-designed prospective studies are required to confirm our findings.

## 1. Introduction

As the predominant endocrine malignancy, thyroid cancer (TC) constitutes 1% of solid organ malignancies; in addition, at 9%, it has a higher incidence in women [1]. TC contributes to 0.5% of overall cancer-related deaths and is expected to become the fourth leading malignancy worldwide [1]. This increase in its incidence may be related to improvements in diagnostic radiological modalities [2]. In particular, half of differentiated TC (DTC) cases are initially detected by radiological studies [2].

The clinical presentations of TC range from indolent tumors with a low risk of mortality to aggressive tumors, such as anaplastic TC [3]. TC can arise from two types of cells: follicular thyroid and parafollicular cells. DTC (such as papillary TC [PTC] and follicular TC) and undifferentiated TC (such as anaplastic TC and poorly differentiated TC) are examples of cancers that originate from follicular thyroid cells, whereas medullary TC arises from parafollicular cells. DTC accounts for 95% of all cases of TC; of these, PTC is the most frequent type and has a favorable prognosis [3,4]. In contrast, follicular TC, Hürthle cell TC, and poorly differentiated TCs are high-risk malignancies associated with hematogenous metastasis to distant organs such as bone and lung [3].

Environmental factors (including exposure to ionizing radiation, artificial chemicals, water pollution, and cold climates), comorbidities (including obesity, iodine deficiency, insulin resistance, diabetes, and metabolic syndrome), lifestyle factors (including physical inactivity and smoking), biological factors, and diet may increase the risk of TC [1,4,5].

Obesity, a chronic low-grade inflammatory disease, poses a significant health risk and negatively impacts physical well-being at all levels. Body mass index (BMI) is generally accepted as a measure of body fat, wherein overweight refers to a BMI of 25.0–29.9 kg/m^2^ and obesity to a BMI ≥ 30 kg/m^2^ [4]. Notably, the prevalence of overweight and obesity is increasing substantially worldwide, and combined, they are linked to 25% and 50% of all malignancies detected in men and women, respectively [6]. After smoking, obesity is the second most prevalent, modifiable, and preventable risk factor for cancer [4]. Both obesity and overweight have been identified as risk factors for the development of TC [7], and the involvement of obesity has been reported in the occurrence and progression of TC and benign nodular thyroid disease [8]. In particular, a 5-point elevation in BMI increases the risk of TC by 30%, whereas a 0.1-point elevation in the waist-to-hip ratio increases the risk by 14% [9]. The most common TC types observed in individuals with obesity are papillary, follicular, and anaplastic. In contrast, obesity is correlated with a low risk of medullary TC, suggesting a potential correlation with the type and histological specificity of the tumor [4].

The biological pathways linking TC and adiposity remain unknown, although some complex mechanisms whereby excess adiposity promotes cancer initiation and progression have been elucidated. Chronic inflammation, hyperinsulinemia, insulin resistance, intestinal microbiome alterations, disturbances in circadian rhythms, hormone biosynthesis, hormonal pathway flaws, and changes in circulating leptin and adiponectin levels are examples of such mechanisms [7,8]. Notably, excessive food consumption increases leptin levels, a molecule responsible for obesity, which increases the frequency of TC [10].

In Saudi Arabia, TC ranks as the most frequent endocrine cancer detected in women and the ninth most prevalent malignancy among men [10,11]. According to a recent study, the risk of TC in Saudi Arabia increased in both men and women between 1990 and 2019, amounting to 22- and 15-fold increases, respectively [11]. The reasons for this are unknown; however, a family history of TC, iodine deficiency, history of radiation exposure, smoking, obesity, and excessive leptin levels have been reported as possible causes [10,11]. Thus, TC is a substantial health care burden; moreover, it is typically detected in advanced stages owing to the lack of screening guidelines in Saudi Arabia [10]. Furthermore, 50% of the adult population in Saudi Arabia is overweight, and one in every five individuals is obese; consequently, the prevalence of obesity is alarming. As well-known biological risk factors for cancer, diabetes, and cardiovascular diseases, obesity and overweight cause significant disabilities and deaths in Saudi Arabia and worldwide [12].

Thyroid nodules are an often-encountered surgical issue that necessitates thorough evaluation and exclusion of malignancy. Thus, they are evaluated clinically, cytologically, and radiologically. Although fine-needle aspiration cytology (FNAC) is an accurate diagnostic tool, it occasionally exhibits uncertain cytology, which is commonly referred to as atypia of undetermined significance/follicular lesion of undetermined significance (AUS/FLUS). These diagnoses are clinically problematic and difficult to categorize as either benign or malignant [13,14]. Notably, the diagnostic rate of AUS/FLUS should not surpass 7%, as higher rates may suggest overuse of this specific category, particularly when alternative interpretations are more suitable. In the context of AUS/FLUS, the reported risk of malignancy (ROM) ranges from 5% to 15%. However, the ROM reported by different institutions vary significantly, with estimates ranging from 6% to 48% [13,14]. These discrepancies may be explained by the considerable challenges in determining the ROM due to the limited number of AUS/FLUS nodules that undergo surgical intervention. Overestimation of the ROM occurs because of selection bias when relying exclusively on histopathology data for measurement. Conversely, if we make the assumption that nodules that have not been excised are benign, the ROM is inaccurately low when computed using the total number of specimens classified as AUS/FLUS (independent of the surgical follow-up) as the denominator [14]. Therefore, in the setting of AUS/FLUS cases, the diagnostic process for effectively managing the risk of both excessive and insufficient treatment continues to be a subject of controversy [14]. Hence, given the correlation between obesity and TC, the purpose of this study was to explore the impact of BMI on the risk of developing TC in thyroid nodules categorized as AUS/FLUS.

## 2. Materials and Methods

### 2.1. Study Design

This retrospective, single-institution, cohort study was conducted after obtaining approval from the Office of Research Affairs at King Faisal Specialist Hospital & Research Center, Riyadh, Saudi Arabia (publication number 2235139). The requirement of informed consent was waived due to the retrospective nature of this study and because personal data used in this study will not be disclosed.

### 2.2. Patient Data

For the present analysis, we used the dataset previously collected and published by our research team [13]. Data were extracted from electronic medical records, and any missing information was extracted from patient charts. The dataset included all thyroidectomies performed at our center wherein the condition had been diagnosed cytologically as AUS/FLUS within the time frame of January 2011 to December 2014. The Bethesda System for Reporting Thyroid Cytopathology [14] was used to determine all AUS/FLUS diagnoses. The inclusion criteria comprised the availability of the following parameters: patient demographics, BMI at the time of cytological diagnosis, nodule size, and final pathology type (benign vs. DTC). Overall, 113 cases were included, and two were excluded because the final pathological diagnosis indicated lymphoma.

Patients were classified into four categories based on the current BMI definitions: <18.5 kg/m^2^ (underweight), 18.5–24.9 kg/m^2^ (normal weight), 25–29.9 kg/m^2^ (overweight), and ≥30 kg/m^2^ (obesity). Further analyses were also performed by dividing patients into the following two categories: obese (BMI ≥ 30 kg/m^2^) and non-obese (BMI < 30 kg/m^2^).

Nodule size was categorized as follows, according to size thresholds obtained from the American staging system guidelines for TC [15]: ≤10, 11–20, 21–40, and >40 mm.

### 2.3. Aim

The relationship between the ROM and BMI was examined for all data, with subgroup analyses for nodule size, sex, and age.

### 2.4. Statistical Analyses

The Statistical Package for the Social Sciences (version 22.0; SPSS Inc., Chicago, IL, USA) was used for the analyses. The results are presented as median (interquartile range) or the mean ± standard deviation (SD). The association between TC diagnosis and BMI was studied in the entire population. The clinical and radiological features of patients with malignant or benign nodules were compared, and the association between the BMI groups stratified by age and sex was analyzed. Pearson’s chi-square or Fisher’s exact tests were used to compare categorical variables, and Student’s *t*-test, analysis of variance, or the Mann–Whitney test were performed to compare continuous variables. Furthermore, a multiple binary logistic regression analysis generated odds ratios (OR) with 95% confidence intervals (CI) to determine the effect of BMI on the risk of TC. BMI was evaluated as both categorical and continuous. Other factors, including age, sex, and lesion size, were used as confounding variables. All tests were two-sided, and statistical significance was set at *p* < 0.05.

## 3. Results

A total of 113 patients were included in the analysis; of these, 40 had combined AUS and FLUS, 39 had only AUS, and 34 had only FLUS. The mean age of the patients was 41 ± 11.5 years, and 36.3% [41/113] were aged ≥45 years. Most patients were women (*n* = 86, 75.4%). The final pathological results indicated benign tumor in 61 patients (54%) and malignant in 52 (46%). In the malignant group, PTC comprised 94.2% of cases, whereas follicular TC comprised only 5.8%. The mean age was comparable in the malignant and benign groups (41 ± 11.6 vs. 39.9 ± 11.7 years; *p* = 0.62, mean difference 2.0, 95% CI: −2.5–6.5). Of the 113 patients, 50 (44.2%) were obese and 39 (34.5%) were overweight.

The clinical and radiological features of patients with benign and malignant thyroid nodules are presented in Table 1. In men, malignant nodules were more common than benign nodules (32.7% vs. 16.4%; *p* < 0.05).

A total of 46 patients (40.7%) underwent thyroidectomy after receiving a consistent cytological diagnosis of AUS/FLUS in repeated FNAC, whereas the remaining patients (59.3%) had a sole diagnosis of AUS/FLUS. The decision to proceed with surgery in these remaining patients was based on various factors, including clinical assessment, presence of pressure symptoms (such as dyspnea and dysphagia), extent of retrosternal involvement, worrisome findings on neck ultrasonography, lack of response to medical treatment in Graves’ disease, progressive enlargement of the nodule, and patient preference.

The mean BMI was not significantly different between the benign and malignant groups: 29.7 ± 5.8 vs. 29.3 ± 6.2 kg/m^2^ (mean difference 0.40, 95% CI: −1.8–2.6, *p* = 0.50). Furthermore, the mean nodule size was not significantly different between the two groups (benign vs. malignant, 3.5 ± 2.2 vs. 3.3 ± 2.4 cm). The ROM was higher for nodules sized 1.1–2 cm (36.5%), followed by those sized >4 cm (28.8%); however, no significant association was observed.

Two potential risk factors for TC (age and nodule size) were examined, and their associations with malignancy were compared stratified by sex. Overall, the crude and adjusted analyses of the risk factors included data from all 113 patients. Table 2 presents the estimated crude and adjusted OR with age and nodule size as confounders. After controlling for confounding variables, the multiple logistic regression models using all data indicated that BMI was not correlated with an increased ROM (OR 0.99, 95% CI: 0.93–1.06, *p* = 0.87). We obtained similar results when the data were stratified by sex. The adjusted OR was 1.05 (95% CI: 0.9–1.3, *p* = 0.57) for men and 0.99 (95% CI: 0.92–1.06, *p* = 0.82) for women.

The full model of the multiple logistic regression for malignancy is presented in Table 3. Sex was a significant factor after adjusting for age and BMI. Malignancy was two times more likely to occur in men than in women (OR = 2.8, 95% CI: 1.13–7.23, *p* = 0.02).

Table 4 shows the relationship between sex and ROM when the data were subgrouped according to BMI as a binary variable (categorized into non-obese [<30 kg/m^2^] and obese [≥30 kg/m^2^] groups). The ROM was higher in obese men than in obese women (54% vs. 41%); however, the difference was not significant (*p* = 0.4). In populations with BMI <30 kg/m^2^ (i.e., the non-obese group), malignant cases were significantly more common in men than in women (71% vs. 41%; *p* = 0.04).

Obese patients were further subgrouped to test whether there was an association between age and ROM in obese men or women, and no significant difference was noted (*p* = 0.13 and *p* = 0.61, respectively). In addition, the ROM was higher in obese participants aged <45 years compared to those ≥45 years (Table 5).

**Figure 1:**
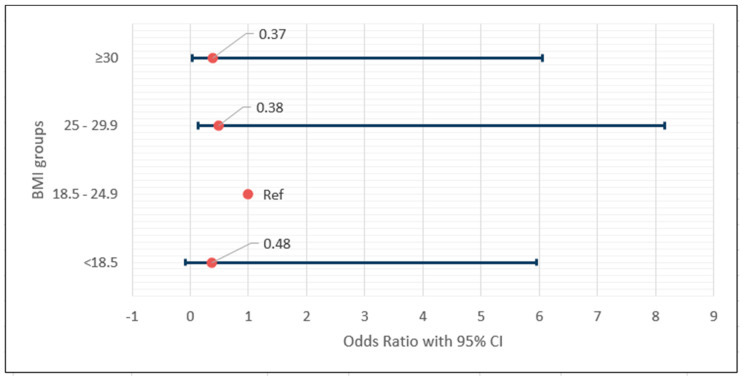
Adjusted odds ratio (95% CI) of the BMI groups.

Figure 1 illustrates the OR of the four BMI groups, with the ORs adjusted for age, sex, and nodule size.

## 4. Discussion

Indeterminate thyroid nodules have differing clinical and radiological features and, as reported previously by our research team, these features could not help in predicting cancer in AUS/FLUS nodules [13,16]. Moreover, we showed that the American College of Radiology Thyroid Imaging Reporting and Data System guidelines were unable to predict malignancy in these cases [16]. Hence, the present study aimed to explore the role of overweight and obesity on the ROM in AUS/FLUS nodules.

A meta-analysis of seven cohort studies with 5154 TC cases confirmed the correlation of obesity and overweight with the development of PTC, although no significant association was found between overweight and the risk of TC among women or Asian participants in the subgroup analyses [17]. The following factors may help to interpret these findings to some extent. First, the utilization of varying BMI reference values resulted in differing prevalence estimations for the overweight category across the diverse populations. Second, in comparison to Caucasians, individuals of Asian descent typically have smaller body frames. Lastly, the prevalence and incidence of TC vary across different countries globally. Moreover, the impact of BMI on the susceptibility to TC in Asian populations may differ from that observed in non-Asian populations [17].

Several theories have been proposed to explain the impact of excessive body weight on TC. Initially, the probable biological mechanisms underlying the link were thought to involve elevated levels of endogenous hormones (estrogens, adipokines, insulin, steroid hormones, and insulin-like growth factor-1) in individuals who are overweight or obese. Additionally, the development of TC involves factors such as the nuclear factor κB system and oxidative stress [17].

In terms of biological mechanisms, adiponectin, an adipokine released by adipose tissue, is a factor that is predominantly expressed in adipocytes and has a crucial role in enhancing insulin sensitivity. Reduced levels of plasma adiponectin have been shown to be correlated with the presence of insulin resistance [4]. Additionally, lower concentrations of adiponectin have been documented in several clinical conditions including atherosclerosis, diabetes, and obesity. Moreover, the effects of adiponectin on tumor tissues are mediated by various intracellular pathways, modulation of tumor angiogenesis, insulin sensitivity, and inflammation [4].

Leptin, a hormone synthesized mainly by adipose tissue and to a lesser extent by other tissues such as those of skeletal muscle and the stomach, has also been proposed as a potential biological mechanism for the correlation between increased body weight and TC [4]. In conjunction with ghrelin, leptin plays a crucial role in the regulation of energy balance and body weight, while also serving a significant function in the storage of energy. In contrast to adiponectin levels, leptin levels have been shown to be positively correlated with BMI, whereby individuals with a larger body fat mass and/or higher BMI tend to have higher levels of leptin. In addition, leptin resistance and increased leptin levels are frequently observed in individuals with obesity. Furthermore, the phenomenon of increased expression of leptin and its receptor has been observed in numerous types of cancers, including TCs [4]. Of note, obesity is frequently associated with reduced insulin sensitivity, resulting in the stimulation of insulin production by pancreatic beta-cells and the development of hyperinsulinemia and insulin resistance, which increase the risk of TC occurrence. The initiation of insulin resistance can be influenced by many variables, such as obesity and diabetes, epigenetic and genetic factors, or exposure to endocrine-disrupting chemicals. In addition, insulin resistance is correlated with high levels of tumor necrosis factor-α. The presence of these various conditions has the potential to elevate the risk of TC [4].

Insulin-like growth factors hold considerable significance in both physiological and pathological contexts. In terms of TCs, they exhibit a positive correlation with the development of DTC. Furthermore, insulin-like growth factor binding proteins reportedly have a potential tumor suppressing effect on DTC [4].

Notably, obesity is linked to a state of persistent low-grade inflammation characterized by non-specific immune system activation and an elevation in inflammatory markers. This condition is accountable for the emergence of numerous clinical disorders associated with obesity, such as hyperglycemia, insulin resistance, hyperinsulinemia, vascular impairment, and hyperlipidemia [4]. All these conditions are correlated with heightened oxidative stress, which is a state in which there is an abundance of reactive metabolites and free radicals that have detrimental effects on the organism. Although oxidative stress has a role in the emergence and advancement of diverse forms of malignancy, its precise impact on TC remains uncertain. However, an association between reduced antioxidant system function and the overproduction of reactive oxygen species has been proposed. Another factor could be a decrease in the expression levels of the antioxidant molecules in TC cells [4].

Estrogens are a class of steroid hormones primarily responsible for regulating the growth, differentiation, and function of reproductive organs. Moreover, these compounds demonstrate a wide array of biological effects on bone, as well as on the cardiovascular and immunological systems [4]. Globally, the prevalence of TC and obesity is higher in women than in men, and this could potentially be attributed to levels of endogenous estrogens, which have the ability to function as growth factors for both malignant and benign thyroid nodules. The process of synthesizing or converting endogenous sex hormones is facilitated by adipose tissue via the action of aromatase, the levels of which are reported to be higher in individuals with obesity. Consequently, estrogens levels are elevated, resulting in an imbalance between estrogens and androgens and leading to the development of TC [4]. 

Furthermore, a positive correlation between BMI and the size of the thyroid gland is noteworthy. As a result, individuals with higher BMIs may have a larger thyroid volume, potentially accommodating a greater number of cells susceptible to mutation, which may subsequently lead to malignancy [17].

A study of 253 patients with indeterminate thyroid nodules found that a higher BMI was associated with lower ROM across all participants except for women aged >45 years [18]. Furthermore, Rotondi et al. [19] found that obese women had a significantly lower rate of suspicious or malignant nodules (Thy4/5). Conversely, another study reported that obese women were more likely to be diagnosed with TC than obese men, with a greater risk in those aged >50 years than in the younger obese population [20]. Our findings revealed that the overall ROM was higher in obese men than in obese women, and the risk was higher in obese men aged <45 years. In addition, Kim et al. [21] emphasized that BMI and body surface area were not prognosticators of PTC behavior in men. However, in women, both these factors were independent predictors of multiplicity. The differences in the risk and behavior of TC between obese men and women could be attributed to hormonal effects and metabolic changes [22].

Similar to our study, other reports found no association between obesity and DTC [19,23]. Moreover, a recent study found no link between obesity and TC or benign nodular thyroid disease [24]. Interestingly, Wang et al. [25] found a higher risk of extrathyroidal extension in overweight and obese patients diagnosed with PTC, and more severe tumor, node, and metastasis (III/IV) staging in the overweight patients. Furthermore, a positive correlation was found between BMI and PTC [25]. Notably, Kim et al. [21] found that BMI was an independent prognosticator of extrathyroidal extension. Our study did not examine such details of the pathological reports, which is one of its limitations.

A large-scale case–control study found that obese or overweight adolescents had a higher risk of PTC during adulthood, implying that controlling adolescent weight is critical in lowering the risk of PTC [26]. Conversely, our data found no statistical significance between the age of obese patients and ROM.

A recent study concluded that bariatric surgeries reduce the risk of cancer by inhibiting adipose-related inflammatory mechanisms, which have a role in tumor initiation and progression [27]. Furthermore, Boru et al. [28] found that eight of 43 patients referred to a bariatric center developed TC, indicating that TC was the second most common cancer in their group. This suggests that morbidly obese individuals are more likely to develop TC, which warrants further investigation.

The link between BMI and the risk of PTC has been reported to be closely related to large lesions (>4 cm), implying that excess adiposity directly affects the growth and progression of DTC [7]. In the same study, a significant association was found between BMI and the risk of anaplastic TCs, highly fatal undifferentiated tumors, which seem to develop as a natural progression of untreated DTC. Other studies have confirmed that no relationship exists between BMI and small PTCs; moreover, detecting such nodules in obese populations via palpation or imaging is difficult [29,30]. Zagzag et al. [2] reported that BMI did not aid in the initial detection of DTC. In addition, they believed that any link between DTC and obesity was unrelated to detecting these lesions. Similarly, our data showed that nodule size was unrelated to a higher ROM in any BMI category.

One of the strengths of our study is that it is one of the first to show no relationship between obesity and TC in AUS/FLUS nodules. Furthermore, it is worth noting that all patients included in the current study possessed well-documented final histopathology reports. However, this study has some limitations, including its relatively small sample size and retrospective nature, which inherently introduces biases. Moreover, the current sample comprised only resected nodules. Consequently, this may result in an overestimation of the ROM and does not accurately reflect all AUS/FLUS nodules, thereby introducing a potential selection bias. Future investigations where lower rates of malignancy are found in patients with AUS/FLUS could better clarify these findings. Lastly, meaningful data that may have influenced our results, such as the location of the patients’ residence, prior use of thyroid medications, details from the pathological reports including Hashimoto’s thyroiditis, and levels of anti-thyroid antibodies (thyroid peroxidase antibodies and thyroglobulin antibodies), were lacking. Further multi-center studies investigating these factors are warranted.

## 5. Conclusions

Our study showed no relationship between obesity and TC in AUS/FLUS nodules. In addition, men had more malignant nodules than benign nodules. Given the complexity of this topic, further well-designed prospective studies are required to confirm our findings.

## Figures and Tables

**Table 1:** Comparison of clinical and radiological features of patients with benign or malignant pathology.

Variables		Pathology			
	Total (N = 113)	Benign (N = 61)	Malignant (N = 52)	*p*-Value	OR (95% CI)
Age, years					
<45	72	37 (60.7%)	35 (67.3%)		1
≥45	41	24 (39.3%)	17 (32.7%)	0.47	0.75 (0.34–1.65)
Sex					
Male	27	10 (16.4%)	17 (32.7%)		1
Female	86	51 (83.6%)	35 (67.3%)	0.04	0.40 (0.16–0.98)
BMI, kg/m^2^					
<18.5	3	1 (1.6%)	2 (3.8%)	0.64	0.55 (0.04–7.03)
18.5–24.9	21	10 (16.4%)	11 (21.2%)		1
25–29.9	39	22 (36.1%)	17 (32.7%)	0.45	0.38 (0.03–4.6)
≥30	50	28 (45.9%)	22 (42.3%)	0.45	0.39 (0.03–4.6)
BMI, kg/m^2^					
<30	63	33 (54.1%)	30 (57.7%)		1
≥30	50	28 (45.9%)	22 (42.3%)	0.70	0.89 (0.41–1.82)
Nodule size, cm					
≤1	12	7 (11.9%)	5 (9.6%)		1
1.1–2	32	13 (22.0%)	19 (36.5%)	0.29	2.0 (0.53–7.8)
2.1–4	34	21 (35.6%)	13 (25%)	0.83	0.86 (0.22–3.3)
>4	33	18 (30.5%)	15 (28.8%)	0.82	1.2 (0.30–4.4)

*p*-values and ORs were estimated using univariate logistic regression. CI, confidence interval; OR, odds ratio; BMI, body mass index.

**Table 2:** Crude and adjusted odds ratios for pathology type by BMI groups and stratified by sex.

	Total (n = 113)				Men				Women			
	Crude OR (95% CI)	*p*-Value	Adjusted OR ^a^ (95% CI)	*p*-Value	Crude OR (95% CI)	*p*-Value	Adjusted OR ^b^ (95%)	*p*-Value	Crude OR (95% CI)	*p*-Value	Adjusted OR ^b^ (95% CI)	*p*-Value
BMI continuous	0.98 (0.92–1.05)	0.73	0.99 (0.93–1.06)	0.87	0.91 (0.85–1.2)	0.91	1.05 (0.9–1.3)	0.57	0.98 (0.91–1.05)	0.69	0.99 (0.92–1.06)	0.82
BMI categories, kg/m^2^												
<18.5	0.55 (0.04–7.0)	0.64	1.5 (0.1–24.1)	0.73	Ref		Ref		0.38 (0.029–5.2)	0.47	0.32 (0.02–5.4)	0.43
18.5–24.9	Ref		Ref		-		-		Ref		Ref	
25–29.9	0.39 (0.03–4.6)	0.45	0.68 (0.21–2.01)	0.51	0.50 (0.03–6.6)	0.60	0.56 (0.03–11.1)	0.72	0.28 (0.02–3.5)	0.33	0.4 (0.024–4.9)	0.43
≥30	0.39 (0.03–4.6)	0.45	0.76 (0.26–2.24)	0.62	0.29 (0.02–3.3)	0.32	0.82 (0.04–14.0)	0.89	0.34 (0.02–4.1)	0.39	0.33 (0.02–4.8)	0.42

^a^ Adjusted odds ratios were estimated using multiple logistic regression, including age, sex, and nodule size (cm). ^b^ Adjusted odds ratios were estimated using multiple logistic regression with the inclusion of age and nodule size (cm) and stratified by sex. OR, odds ratio; CI, confidence interval; BMI, body mass index.

**Table 3:** Full model of multiple logistic regression for malignancy with sex, age, and BMI as predictors.

Factor	Group	B	S.E.	*p*-Value	Odds Ratio	OR 95% CI	
						Lower	Upper
Sex	Female				1		
	Male	1.05	0.473	0.026	2.859	1.13	7.23
Age, years	≥45				1		
	<45	0.433	0.422	0.305	1.542	0.675	3.524
BMI, kg/m^2^	18.5–24.9			0.774	1		
	<18.5	−0.691	1.313	0.599	0.501	0.038	6.571
	25–29.9	−1.011	1.283	0.43	0.364	0.029	4.494
	≥30	−1.066	1.27	0.401	0.344	0.029	4.147
	Constant	0.26	1.295	0.841	1.297		

BMI, body mass index; B, Beta estimates; S.E., standard error.

**Table 4:** Association between sex and risk of malignancy when BMI is <30 or ≥30 kg/m^2^.

	<30 kg/m^2^ (n = 63)			≥30 kg/m^2^ (n = 50)		
	Pathology			Pathology		
Sex	Benign	Malignant	*p*-Value *	Benign	Malignant	*p*-Value *
Male	4 (29%)	10 (71%)	0.04	6 (46%)	7 (54%)	0.4
Female	29 (59%)	20 (41%)		22 (59%)	15 (41%)	

* *p*-value estimated by independent Chi-square test; raw percentages were used to estimate the risk of malignancy.

**Table 5:** Association between age and risk of malignancy in obese (BMI ≥30 kg/m^2^, n = 50) patients.

	Obese Males (n = 13)			Obese Females (n = 37)		
	Pathology			Pathology		
Age, in years	Benign	Malignant	*p*-Value *	Benign	Malignant	*p*-Value *
<45 (n = 5)	1 (20%)	4 (80%)	0.13	16 (57%)	12 (43%)	0.61
≥45 (n = 8)	5 (63%)	3 (38%)		6 (67%)	3 (33%)	

* *p*-value estimated by independent Chi-square test; raw percentages were used to estimate the risk of malignancy.

## Data Availability

The data presented in this study are available on request from the corresponding author.
